# Non-Occupational Post-Exposure Prophylaxis for HIV: 10-Year Retrospective Analysis in Seattle, Washington

**DOI:** 10.1371/journal.pone.0105030

**Published:** 2014-08-20

**Authors:** Sarah J. McDougal, Jeremiah Alexander, Shireesha Dhanireddy, Robert D. Harrington, Joanne D. Stekler

**Affiliations:** 1 Department of Medicine, University of Washington, Seattle, Washington, United States of America; 2 Department of Epidemiology, University of Washington, Seattle, Washington, United States of America; 3 Veterans Affairs Puget Sound, General Medicine Service, Seattle, Washington, United States of America; Commissariat a l'Energie Atomique(cea), France

## Abstract

Despite treatment guidelines in place since 2005, non-occupational post-exposure HIV prophylaxis (nPEP) remains an underutilized prevention strategy. We conducted a retrospective chart review of patients presenting to a publicly-funded HIV clinic in Seattle, Washington for nPEP between 2000 and 2010 (N = 360). nPEP prescriptions were provided for 324 (90%) patients; 83% of prescription decisions were appropriate according to Centers for Disease Control and Prevention guidelines, but only 31% (N = 111/360) of patients were considered “high risk.” In order to use limited resources most efficiently, public health agencies should target messaging for this high-cost intervention to individuals with high-risk HIV exposures.

## Introduction

HIV post-exposure prophylaxis (PEP) is a 28-day course of antiretroviral medication provided to HIV-negative individuals within 72 hours of an exposure to prevent HIV acquisition. In 1995, the Centers for Disease Control and Prevention (CDC) issued the first guidelines for provision of PEP for occupational exposures following a case-control study of healthcare workers which demonstrated that zidovudine reduced the risk of HIV infection by 81% [1], [2]. Studies of HIV-infected pregnant women also demonstrated that timely provision of antiretrovirals could prevent vertical transmission of HIV [3], [4]. In 2005, the CDC issued guidelines for non-occupational PEP (nPEP) after sexual exposures or exposure through shared injection equipment [5], although nPEP had been prescribed in community settings prior to release of these guidelines [6], [7].

Despite the potential benefit, provision of nPEP remains controversial because of the expense (∼$1135/patient [8]), toxicity of PEP medications, and relatively low risk of HIV transmission per exposure. Furthermore, some patients present for care three or more days after exposure. This is problematic as animal models suggest that PEP is only effective if provided within 72 hours after an exposure [9]–[11].

Historically, most nPEP has been provided to men who have sex with men (MSM) following sexual exposures [6], [12]–[14]. MSM are at particularly high risk for HIV acquisition [15] because unprotected receptive anal intercourse (URAI) carries a relatively high rate of transmission per sex act: 0.05%–1.4% for URAI compared to 0.002% for unprotected receptive vaginal intercourse [16], [17]. Despite the high cost of nPEP, model-based cost-effectiveness analyses demonstrate that nPEP after URAI is cost-saving [8], [18], [19]. Given the finite resources for HIV treatment and prevention, proper targeting of nPEP provision is crucial.

Madison Clinic is a Ryan White-funded outpatient clinic operated within Harborview Medical Center (HMC), a county hospital and level one trauma center in Seattle, Washington, that has provided nPEP since early 2000. CDC guidelines recommend that nPEP be provided for “persons with a nonoccupational exposure to blood, genital secretions, or other potentially infected body fluids of a person known to be HIV infected when that exposure represents a substantial risk for HIV transmission and when the person seeks care within 72 hours of exposure” [5]. Using chart record data from nPEP visits at Madison Clinic we sought to: 1) describe the demand for nPEP at Madison Clinic, 2) evaluate prescription decisions against the CDC guidelines as closely as possible given the information available in the clinical records, and 3) evaluate prescription decisions against more restricted guidelines based on findings that nPEP is only cost-effective when provided for URAI exposures [8], [18]. Additionally, we describe the source of funding among persons receiving nPEP to address nPEP program sustainability.

## Methods

We conducted a retrospective chart review of patients evaluated for nPEP at Madison Clinic between April 11, 2000 and November 29, 2010. Demographic characteristics and the following variables were abstracted from electronic medical records for all patients evaluated for nPEP: history of sex with men (for male patients), exposure that precipitated nPEP evaluation, relationship to the source patient, HIV status of source patient, condom use during exposure, baseline HIV status of patient, follow-up HIV status of patient, time from exposure to baseline visit at HMC, dates for baseline and follow-up visits, nPEP regimen and duration of prescription, and CD4 count and viral load for patients found to be HIV positive. nPEP payment information was abstracted separately by HMC pharmacy staff and linked to chart information for each subject. All evaluations were considered separate events; some patients returned for one or more evaluations.

### Ethics Statement

This medical records review was approved by the University of Washington Human Subjects Division. All patient records were de-identified prior to analysis.

### Analyses

We characterized cases as either appropriate or inappropriate for nPEP from a *public health perspective* according to the exposure risk profile and the time interval from the exposure to first contact with HMC. Provision of nPEP was deemed appropriate when: 1) patients were evaluated for nPEP within 72 hours of exposure; AND 2) exposure included receptive or insertive anal or vaginal intercourse or intravenous drug use (IDU); AND 3) no condom was used, condom malfunction occurred or no condom information was available; AND 4) source was known to be HIV-positive or was of unknown HIV status. CDC guidelines for nPEP do not address situations where the source's HIV status is unknown; in these cases prescription decisions are made on a case-by-case basis depending on the HIV seroprevalence of the source's population. Nationwide, HIV seroprevalence among heterosexuals is 2.3% [20] overall and 19% among MSM [21]; in Seattle, WA the HIV seroprevalence among MSM is 19% [22]. Because this data lacked enough information about the source to determine seroprevalence, any source with an unknown HIV status was considered a substantial risk. Provision of nPEP to patients whose exposure was unknown (i.e., missing data), but who indicated that exposure was to an HIV-positive source or a source of unknown HIV status was also considered appropriate. Patients who did not meet all of these criteria were considered inappropriate for nPEP. Many patients experienced multiple exposures (e.g., receptive anal intercourse and insertive oral intercourse during the same event); in these cases, the exposure with greatest risk for HIV acquisition was included in analysis.

Based on recommendations from cost-effectiveness models to prescribe nPEP only for high risk exposures (MSM exposed to HIV via URAI) [8], [18], we further characterized appropriate exposures as “high risk” when: 1) patients were identified as MSM; AND 2) exposure included RAI; AND 3) source was known to be HIV-positive or was of unknown HIV status; AND 4) no condom was used, condom malfunction occurred or no condom information was available; AND 5) patients were evaluated within 72 hours of exposure. Results are presented as descriptive statistics. All data were arranged and analyzed using Microsoft Excel.

## Results

Between April 2000 and November 2010, 360 patients were evaluated for and 324 (90%) were prescribed nPEP (Table 1). Median age was 30 years (range 14–68). HMC's patient assistance program – a hospital-based program that covers the cost of medications for patients who cannot afford them – paid for one third (N = 119/324) of all PEP medications, private or public health insurance covered 34% and patients paid out-of-pocket in 5% of cases. Medication payment information was missing for 24% of patients.

**Table 1:** Presentations for nPEP by exposure risk level.†

Characteristics	Inappropriate^a^	Appropriate^b^	High Risk^c^	Total
	(N = 83)	(N = 166)	(N = 111)	(N = 360)
Provided nPEP	56 (67%)	162 (98%)	106 (95%)	324 (90%)
Gender				
Female	19 (23%)	72 (43%)	0	91 (25%)
Transgender (MtF)	1 (1%)	1 (1%)	1 (1%)	3 (1%)
Male	63 (76%)	93 (56%)	110 (99%)	266 (74%)
MSM	48 (58%)	56 (34%)	111 (100%)	215 (60%)
Age‡				
13–24	16 (19%)	38 (23%)	27 (24%)	81 (23%)
25–34	35 (42%)	62 (37%)	52 (47%)	149 (41%)
35–44	16 (19%)	38 (23%)	17 (15%)	71 (20%)
45–54	10 (12%)	23 (14%)	9 (8%)	42 (12%)
>55	6 (7%)	5 (3%)	6 (5%)	17 (5%)
Race/Ethnicity				
Caucasian	57 (69%)	100 (60%)	71 (64%)	228 (63%)
African-American	8 (10%)	26 (16%)	11 (10%)	45 (13%)
Hispanic	6 (7%)	8 (5%)	10 (9%)	24 (7%)
Asian	2 (2%)	11 (7%)	2 (2%)	15 (4%)
Indian/Middle East	0	2 (1%)	0	2 (1%)
Native American	1 (1%)	3 (2%)	1 (1%)	5 (1%)
Missing	9 (11%)	16 (10%)	16 (14%)	41 (11%)

† Risk levels (inappropriate, appropriate and high risk) here and elsewhere in this manuscript specifically describe a determination of whether nPEP should be provided *from a public health perspective*. Individual providers should make case-by-case determinations for their patients informed by the CDC guidance for nPEP provision.

a Inappropriate risk  = 1) evaluated >72 hours; 2) risk event did not include receptive or insertive anal or vaginal intercourse or intravenous drug use (IDU); 3) used a condom; OR 4) source contact was known to be HIV-negative.

b Appropriate risk  =  patients 1) evaluated for PEP ≤72 hours; 2) risk event included receptive or insertive anal or vaginal intercourse or intravenous drug use (IDU); 3) did not report using a condom or experienced condom malfunction; and 4) source contact was known to be HIV-positive or was of unknown HIV status.

c High risk  =  patients appropriate for nPEP and also: 1) were identified as MSM; and 2) engaged in RAI.

‡ Same as age categories used in CDC HIV Surveillance Report Volume 17, Number 4.

Most patients evaluated for nPEP identified a sexual contact as their HIV exposure (N = 334\360, 92.8%). Sixty percent (215/360) of all patients who were evaluated for nPEP and 59% (191/324) who were prescribed nPEP were MSM. Sexual assault was a factor in 22% (79/360) of all cases and nPEP was prescribed in all but one case. Forty-nine percent (177/360) sought nPEP within 24 hours of exposure and 93% (334/360) reported within 72 hours. Among the 324 patients prescribed nPEP, 214 (66%) were prescribed a 3-drug regimen; 106 (33%) were determined to be high risk, 162 (50%) were appropriate but not high risk and 56 (17%) were inappropriate for nPEP (Table 2). Eighty-nine percent (287/324) of patients prescribed nPEP presumably completed the full 28-day course of medication and the remaining 11% (37/324) are known to have stopped early. Fifty-three percent (172/324) of patients never returned for a follow-up visit. Of the patients who did return for a follow-up visit, 39% (126/324) returned within 8 weeks and 2% (5/324) returned by 12 weeks following initiation of nPEP. Five percent (15/324) returned for an HIV test more than three months following initiation of nPEP.

**Table 2:** Risk behaviors among patients exposed to HIV-positive contact or contact with unknown HIV status (N = 351).*

Source Contact	HIV Positive	Unknown Status	
Status	173 (48%)	178 (49%)	
Source Contact†	Provided PEP/Request PEP	Provided PEP/Request PEP	Total
Regular	39/43 (91%)	5/6 (83%)	49 (14%)
Casual	75/82 (91%)	42/46 (91%)	128 (36%)
Anonymous	30/32 (94%)	99/106 (93%)	138 (39%)
Comm Sex Worker	0	5/5 (100%)	5 (1%)
Missing‡	11/16 (69%)	15/15 (100%)	31 (9%)
Exposure Type	Provided PEP/Request PEP	Provided PEP/Request PEP	Total
Receptive Anal	61/64 (95%)	65/73 (89%)	137 (39%)
Insertive Anal	42/46 (91%)	21/22 (95%)	68 (19%)
Recept/Insert Vag	27/29 (93%)	52/54 (96%)	83 (24%)
Recept/Insert Oral	13/19 (68%)	4/4 (100%)	23 (7%)
IV Drug Use	3/3 (100%)	10/10 (100%)	13 (4%)
Needle stick/other	7/10 (70%)	2/3 (67%)	13 (4%)
Missing	2/2 (100%)	12/12 (100%)	14 (4%)
Condom Use	Provided PEP/Request PEP	Provided PEP/Request PEP	Total
Yes	6/7 (86%)	5/5 (100%)	12 (3%)
Broke/Slip/Remove	46/51 (90%)	33/37 (89%)	88 (25%)
No	86/95 (91%)	84/90 (93%)	185 (53%)
Missing	17/20 (85%)	44/46 (96%)	66 (19%)

*Nine (2.5%) patients were excluded from this table because they reported that their source contact was HIV-negative or because data about their source contact was missing.

† Four (1%) patients reported that his/her contact was HIV negative: one reported a regular source contact, one reported an anonymous source contact, and data for source contact type was missing for two of these patients.

‡ Information about source contact was missing for five (1.5%) patients.

Three MSM patients (0.9%) tested positive for HIV when they presented for nPEP, and four patients (1.2%) seroconverted after their baseline visit. Two of these cases are potential nPEP failures as they tested HIV positive at two and five months following PEP, respectively. One additional patient tested negative at his baseline visit and at 11 days following the completion of nPEP, but tested HIV positive at five months indicating a potential nPEP failure. And one patient is unlikely to be an nPEP failure as he tested HIV negative as late as 1 year after receiving nPEP. Twenty-six of 326 patients (8%) sought nPEP at Madison Clinic at two or more different times (N = 21) or reported a previous nPEP visit at another clinic (N = 5).

Among patients who received nPEP at Madison Clinic, 83% (268/324) met CDC guidelines for provision of nPEP. Of those reporting a high-risk exposure to HIV (MSM with URAI), 95% (106/111) were prescribed nPEP. Ninety-eight percent (162/166) of patients who were appropriate for nPEP, but not considered high-risk, were prescribed nPEP and 67% (56/83) with exposures inappropriate for nPEP received a prescription. Exposures for which nPEP was prescribed inappropriately from *a public health perspective* included high risk exposures (e.g., URAI) that occurred >72 hours before presentation for care, oral intercourse, exposure to an HIV-negative source and lower-risk exposures (e.g., ingestion of breast milk by an adult, semen contact to hangnail).

## Discussion

This study describes provision of nPEP through a program embedded within the HIV clinic of a publicly-funded urban hospital. Only 360 people were evaluated in the 10 years of the Madison Clinic nPEP program. While an estimated 41,000 MSM live in King County [23] and 2,091 MSM were diagnosed with HIV between 2001 and 2010 in King County [24], only 111 MSM sought nPEP at Madison for high-risk exposures (i.e., MSM with URAI exposures) during this time. In these clinical data, nPEP was underutilized by those who would benefit the most from it and perhaps provided too often to patients whose risk for HIV infection is too low to outweigh the cost and risks of the medication, given their exposure.

Our results are consistent with previously published literature demonstrating low utilization of nPEP. Surveys of MSM in U.S. and European cities find that this high-risk group is poorly informed or unaware of nPEP programs [25]–[27]. In surveys conducted among attendees at the Seattle Gay Pride parade (2009–2012) only 26% of attendees were aware of nPEP [unpublished data, Elizabeth Barash]. The majority of studies, with a few exceptions [6], [28], report low demand for nPEP [13], [29]–[31]. In order to access and benefit from nPEP, individuals must overcome many barriers: 1) recognize an exposure to HIV; 2) be aware of nPEP and where to obtain it; 3) report for care within 72 hours of exposure; 4) have the means to pay for nPEP if they are un- or under-insured and cannot access assistance programs; and 5) preferably know or determine the HIV status of her/his contact. These are difficult for community members to accomplish without clear, consistent and abundant nPEP education. Despite these challenges, public health programs are beginning to include nPEP in their prevention programs, such as the M*SHP minus 36:00 nPEP program implemented in New York City to provide free nPEP [32].

If nPEP programs are to be sustainable and have a public health impact, clinicians may need to limit nPEP prescription to those exposures for which nPEP is cost-effective. Pinkerton *et al* [18] found that nPEP is cost-saving for MSM recently exposed to HIV through URAI, but no other exposure was either cost-saving or highly cost-effective (≤$60,000/QALY). We found that 67% (56/83) of patients with an exposure that did not meet the CDC's nPEP guidelines received a prescription and that 36% (20/56) of these were covered by HMC or the patient assistance program. If one third of nPEP prescriptions are funded with public health dollars, nPEP programs might not be sustainable or effective unless they can be limited to HIV exposures with the highest risk. nPEP should be judiciously prescribed according to CDC guidelines and with cost-effectiveness in mind. However, we acknowledge that clinicians faced with a frightened patient and vague guidelines often prescribe nPEP treatment in cases of low transmission risk, illustrating the difficulty of caring for patients exposed to HIV [33], [34]. Clinicians should also consider the risk profile of each patient as even generally low-risk exposures, such as oral sex, can present higher risk for HIV under certain circumstances.

Our study has several limitations. Although we identified three potential nPEP failures among four seroconversions, it is possible that we failed to identify additional nPEP failures because of the high rate of loss-to-follow-up. Additionally, information regarding patient behavior, such as reported condom use, was determined through self-report and is subject to recall bias. Similarly, the HIV status of the patients' contacts was also patient-reported and could be inaccurate for several reasons including dishonesty or unknown recent HIV acquisition by the source [35], [36]. Data regarding follow-up with primary care providers and treatment adherence were not available and Madison providers often encourage patients seeking nPEP to follow up with their primary providers, indicating that our records may underestimate follow-up rate for these patients. Similar poor rates of follow-up have been observed in other studies of both sexual assault victims (41–56% follow-up) [33], [37] and others (34% follow-up) [28]. Finally, because we present data only for persons seeking evaluation for nPEP at the county hospital, this study may not be representative of all exposures that occur in the Seattle area population.

## Conclusions

In summary, nPEP and now pre-exposure prophylaxis (PrEP) are potentially powerful prevention methods alongside other effective methods such as condoms and frequent HIV testing. In order for nPEP to have a population-level impact on HIV prevention, education and promotion must be intensified and targeted to groups who would benefit most from nPEP: MSM who have unprotected receptive anal intercourse. Establishing sites for the provision of nPEP has additional benefits, including serving as resources for HIV case-finding and as an entryway into PrEP programs. To be sustainable and socially equitable, nPEP programs will need to take advantage of both public and private funding mechanisms and industry-sponsored patient assistance programs as it is unclear whether the Affordable Care Act will support the provision of nPEP or PrEP.

Patients and clinicians would benefit from more detailed guidelines for provision of nPEP as exposure to HIV is often charged with fear that overwhelms rational assessments of risk. The existence of such guidelines would allay the anxiety of patients and reassure clinicians as they make their recommendations for risk-appropriate use of nPEP.
